# Sunt verba rerum: the pragmatic life of words

**DOI:** 10.1186/s40064-016-3269-z

**Published:** 2016-09-23

**Authors:** Jacob L. Mey

**Affiliations:** 1 Institute of Language and Communication, University of Southern Denmark, Campusvej 55, 5230 Odense, Denmark; 2 1100 West 29th St., Austin, TX 78703-1915 USA

## Abstract

Pragmatics is not about language as such, viewed in isolation, but about words as they are being used. And words are never things, pure objects; words have their history and lives: their story is the story of their users. Pragmatic thinking focuses not just on what ‘is’ there (the ‘essentialist’ method of linguistics), but on how what ‘is’ there, ‘got’ there, and what it ‘does’ there, in a ‘functionalist’ approach, characteristic of pragmatics. Such a functional approach relies heavily on the processes that are material in creating the conditions for words to be used in a particular way: both those processes we normally call ‘historical’ (the history of what has been) and those that are characteristic for what happens in our own times: the pragmatic life of words. I will illustrate these reflections by focusing on a series of well-known linguistic phenomena, first of all the historical ‘emptying’ of content known as ‘semantic bleaching’, and second the transformation of the ways words function, commonly known under the name of ‘grammaticalization’ or ‘grammaticization’. To better understand these processes, I will first focus on what I call the ‘historical bias’ of structuralist linguistics, including a brief discussion of issues having to do with language development and how linguists and historians traditionally have tried to deal with this.

## On history and function

A first observation is that ‘history’ and ‘function’ often are interpreted in different ways; and that in particular, ‘history’ is seen as the history of wars and kings and things linguistic, while ‘functional’ often is narrowly misinterpreted as ‘that which functions within the confines of some grammatical description’. Here Antoine Meillet, the great historical linguist, tells us that such an attitude is basically wrong: history is social; it is more than just the history of things, even if those ‘things’ happen to be words.

Here Virgil’s immortal phrase, which I have adapted slightly to fit my purpose, has it right: *sunt verba rerum*, words are for a reason’, and they have to be spoken for a reason: in other words, they have to have a *function*. Moreover, they should not be spoken, as in Virgil’s story of Aeneas, just ‘to a queen’ (*infandum, regina*…), but to all of humanity, to the great community of language users. (‘Sunt verba rerum: the pragmatic life of words’—freely after Virgil, *Aeneid* I: 462: *Sunt lacrymae rerum et mentem mortalia tangunt* ‘Tears are for a reason: being mortal breaks the heart’; my translation).

This amounts to saying that history, in particular the history of words and their lives, is pragmatic: it takes the users of the words and their society into full account for the description of their functions and the functions of the words they use. Here is what Meillet has to say on this in the *Introduction à l’étude comparée des langues indo*-*européennes* (and had been saying ever since 1902, the year the book first came out):

“La langue n’existe qu’en vertu de la *société*: de même que les sociétés humaines ne sauraient exister sans langage”

‘Language does not exist except through society, just like the human societies could not exist without the use of language’ (1937: 18)

Note here in particular how Meillet carefully distinguishes between ‘*langue’*, the object of linguistic description (a description which for him was mainly historical) and ‘*langage’*, language as it is used, as it functions. The latter is the foundation of society, that is, it is both the historical and the continued precondition of society’s existence; it is also the proper object of pragmatic studies. So let’s make a little excursus into history and see what we can learn about language use from that.

## History of things and history of language

Over the centuries, there have been a number of views on history, both of events and people, and of languages. Just to name a few early trends, along with their language parallels:

- anecdotal (Herodotus’ *Histories*)
  - language parallels: word collecting, word lists, anecdotes etc.
- instructive (Thucydides’ *Peloponnesian War*)
  - language parallel: etymology, exegesis, analogy, educational use, grammatical correctness (rhetors)
- pragmatic (Xenophon’s *Kyroupaideía*, Caesar’s *De bello gallico*)
  - language parallel: discourse, argumentation, rhetoric (Sophists)
- philosophical (Lucretius’ *De natura deorum*, Plato’s myths)
  - language parallel: origin of words (Plato’s *Cratylos*), the ‘real’ meaning of words (like in ‘etymo-logy’, the meaning of the ‘real’, the *etymon*)
- linguistic (Johann Christoph Adelung & Johann Severin Vater’s *Mithridates*, a collection of language specimina, late 1700s-early 1800s)
  - language parallel: history of languages comparing their development as ‘trees’ or families (Jakob Grimm’s *Deutsche Grammatik*; the later comparative linguists: Rasmus Rask, Franz Bopp, August Schleicher and their successors until our own times)

## The history of words

### Documentary, history and story

It is often said that stories are different from documentaries: a story is fiction, a documentary has to do with facts. A movie that is based on facts but poses as a documentary is therefore an historical falsification.

To this, I would say that any writing is to a certain degree a ‘falsification’, in the sense that no writing can equal the facts as they have happened, because all writing (even of documentaries) presupposes a writer. When a story is written, the facts are altered: they are seen through the perspective of the writer and his or her history (or ‘herstory’, as some would prefer to say). The story of the writer overlays and undercuts the story of the history.

The problem here is that while we may be able to see stories as falsified history, we do not see History (with a capital H) as a collection of falsified stories. What happens in nationalistic history-writing is that a story takes over the facts (including the language) and becomes institutionalized as history. I will illustrate this with a piece of historicized story-telling, the (his)story of the American South.

### The story of the south

There is currently a strong tendency to revise the history of the Civil War in America. First, there was of course the official history, of how the slaves were freed and how the North battled the South on humanitarian principles. This history was attacked by Southern writers who glorified the genteel living style of the plantation (think *Gone with the wind*) and stressed the fact that the black people were worse off after 1866 than before (which was partly true).

Subsequently, another deconstruction of history set in, according to which the whole war was a matter of economic interest: the industrialized North needed cheap labor power to compete with the slave economy in the South; the slaves were set free to become the industrial proletariat in cities like Chicago, whereto they were transported by the wagonload from Mississippi, to eke out a miserable existence in the slaughter houses and urban labor camps of their employer, the Armor company.

Without adjudicating claims to correctness (historical or factual) of any particular account, the point to be made here that one cannot write history without creating a story. If successful, then the story becomes history, until it in its turn is superseded by the next successful story. The most recent successful story then becomes History, capitalized. Rather than reviving history and learning from history in order not to repeat it (as George Santayana has taught us), we revise it in our own image to ensure that the repetition does not hurt, as was the case for the American South.

To take some more recent examples, for many people, Mozart is the one who wrote a piece called ‘Elvira Madigan’: they associate his piano concerto #24 with the film whose main musical theme was taken from that work. Similarly, Steven Spielberg’s 2005 film *Munich* will probably become the authorized version of what happened at the Olympic Village in Munich in 1972, when Palestinian terrorists kidnapped and killed eleven Israeli athletes, and themselves got killed in retaliation by the Israeli security forces.

Even the most faithful documentary cannot escape this historical fate. In another Spielberg movie, *Schindler’s List*, even though the film itself was not intended as a documentary, long stretches were purely actual historical footage; however, even these documentary parts became part of the story, and although they represented historical facts, directly so to speak, they were not part of any objective history (as supposedly is the case in a documentary), but of the Spielberg story. For those who viewed the movie, however, Spielberg’s story has become history, because “that’s what happened in Auschwitz, and I have seen it myself”. One is reminded of Ronald Reagan, who once was asked if he would have handled the Iran hostage crisis^1^ different than Jimmy Carter did. His reply was: “I would just have gone in there—I’ve done it many times [read: as an actor].”

Now let’s apply this to the subject of language, and consider how linguists treat facts and historical correctness.

## Historical truth and linguistic facts I: the case of conversation analysis

### Of truth and facts: ‘let the facts speak’

There is a story about an American linguistics professor from the sixties, who sent one of his students to do fieldwork in Central America, among a tribe where he himself had spent some of his formative years. When the student came back, the professor’s first question was: “Did you get the language?”

What this question really is about, is whether the student got everything that counted as a *linguistic fact*. The professor was not particularly interested in what the student had experienced as a ‘displaced person’ in the jungle, nor in what the natives considered a ‘linguistic fact’ (by what is usually called ‘folk linguistics’), nor in what kind of linguistic and other customs the natives might have in dealing with major life events such as births, deaths, and other ‘rites of passage’. The slogan ‘Let the facts speak’ was only applicable to honest-to-God linguistic facts.

With a take-off on the well-known judiciary formula ‘the truth, the whole truth and nothing but the truth’, one could say that the idea of getting ‘the language, the whole language, and nothing but the language’ has been popular among linguists for most of the science’s history (just as its venerable counterpart has been in the courtrooms of justice).

### Facts and conversation

A peculiar variant of this belief in ‘facts’ and this practice of ‘fact-finding’ is encountered in language-based sociological analyses of the conversation analysis (CA) type. Here, if anywhere, the facts are supposed to ‘speak for themselves’: speakers’ utterances are faithfully monitored, and transcribed in great detail; in addition, now that we can easily capture human behavior on video, also gestures and facial expressions are included in those facts. The analyst’s task its to render those facts as faithfully as possible, without introducing any preconceived ideas. Any interpretation should ‘emerge’ from the facts: we only have to give the facts a voice so they can ‘speak for themselves’.

Of course, this is all metaphorical: nobody has ever seen a fact open its mouth and speak. But to enable that fact to ‘speak’ (whatever that means), the voice of the analyst should be muted or even eliminated from the record: he or she is just a conduit, the means by which the fact may be communicated and analyzed, having become transparent in the linguist’s transcription.

What those conversational fact-hunters never seem to realize is that finding their isolated, disembodied facts is not the only, and certainly not the most important part of the description process. Finding ‘facts’ is like taking photographs or stills, a snapshot that freezes the historical movement. But even the most advanced techniques, such as movie or video recording, complete with color and sound, have their own limitations, which are often not realized as such. A movie is not just a collections of stills put together to resemble motion (the original name of the technique of ‘motion pictures’): the pictures themselves are not nearly as important as are the techniques of assembling, sequencing, and cutting the scenes into a film. What we see when watching a movie is not a recording of reality, but an interpretation of some theme or story, offered us through the medium of the pictorial material, arranged in such a way as to reflect the director/moviemaker’s ‘story of the story’ in which we see the facts as obeying a particular order: chronological, final, or causal.

A further important perspective of this composition is that of *choice*. Long before a movie is put together, the original selection has taken place: the moviemaker must determine which of the frames are going to be used where, and which not; in addition, he or she must decide on how each sequence of pictures is going to be put in relation to the other sequences. All these decisions are the director’s alone, and he or she determines what the movie, in the final instance is going to be. In other words, movies are fiction, even if they claim to, or in fact do, represent reality (as in the case of documentaries, pseudo- or real).

Something similar happens in CA, where the analyst has to make a choice among the endless hours of taped (and transcribed) conversation that are at his or her disposal. Here I want to emphasize, first, that the transcription itself is not a *fact* of speech, but an *artifact* of history: no two transcriptions are the same, no matter how hard one tries to standardize the conventions. But even more important is the way of selecting the material to be used in the analysis. This is by no mean an irrelevant or trivial matter: just as the skillful defense lawyer can select and arrange the facts of the case so as to picture the events in a way that is most favorable to his client, so too the conversation analyst ‘proves’ his or her point by selectively choosing among the raw material of transcribed speech. In the case of film making, one could say (varying the old proverb, *C’est le ton qui fait la musique*) that ‘it is the cutting that makes the movie’; similarly, in CA, it’s the selection that makes the analysis. In both cases, we have to take the cutter, respectively the analyst into account; their voices can never be entirely subdued. While pretending to objectively describe and neutrally analyze the historical event of a conversation, what conversation analysis produces is not history, but a story, neither more nor less.

## Historical truth and linguistic facts II: semantic ‘emptying’ (often called ‘bleaching’) and pragmatic misunderstanding

### Semantic ‘bleaching’

But the story does not end here. In the study of language in general (not just in CA), what we are dealing with is not a description of static, historical ‘facts’ or events, but rather, the realization of an historical tendency, a stream of ever-changing events that interlace and embrace, imbricate and reinforce one another. Over time, these events (expressed as they often are in words of a story, or quotes from a source) become solidified in their imbrications, such that it becomes almost impossible to understand them as they were originally meant. But then again, neither is what they originally meant, the same as what they mean today.

Over time, words lose their meaning. Nobody thinks of ‘sails’, when a decidedly sail-less vessel (such as an atomically-powered submarine) is said to be ‘sailing’ from its base in Connecticut to some undisclosed destination in the Middle East. But there are contexts in which an original meaning seems to be kept, while it in reality is lost; the ‘keeping’ masks an act of thievery on the part of an outside agency such as a government, a political party, a religious authority, and so on. This ‘emptying’ of meaning is sometimes called ‘semantic bleaching’: the words lose their colors, fade, as garments do when they are exposed to direct sunlight over time. The next section will give some examples.

### ‘Blessed he who comes in the name of the lord…’

In the Roman Catholic Mass, the chanting of *Sanctus Sanctus Sanctus* ‘Holy Holy Holy’ is followed by the words *Benedictus qui venit in nomine Domini*, usually translated as ‘Blessed he that cometh in the name of the Lord’. The text refers to an episode from the New Testament, where Jesus enters the Holy City with his Apostles on what is now called Palm Sunday. The words are the welcome greetings that the inhabitants of Jerusalem proffered on the occasion of the triumphant entry of one they thought might be the long-awaited Messiah; the words ‘Blessed he that cometh in the name of the Lord’, as found in St. Matthew 21:9 (King James Version) are actually themselves a quote from the Old Testament, where they occur in Psalm 118:26. In this way, the invocation has been doubly or triply codified as belonging to the sacred sphere of Church and Scripture. And this is precisely where pragmatic misunderstandings due to ‘bleaching’ come into the picture. Consider the following.

On entering an airplane belonging to Continental Airlines and after being seated, one used to be treated to various linguistic variants of the standard formula ‘Welcome aboard’. The different languages are ranked alphabetically, and when the letter H comes up, we see a Hebrew display with the text *Barukhim Ha*-*ba’im’* ‘Welcome they who (have) come’. Except for the part that is missing, ‘in the name of the Lord’ (which would be slightly less suitable in the present situation), the text is identical to the one in Matthew 21.

Here’s another example. On entering the settlement Tel Qatifa, which used to be situated in the Gaza strip prior to its forced evacuation in 2005, one was greeted by a big sign saying *Barukhim ha*-*ba’im leTel*-*Qatifa* ‘Welcome to Tel-Qatifa’. Again, the literal translation would be very similar to the one used in the religious context: ‘Blessed they who have come’, but evidently no such connotation was present in the minds of the settlers who put up the sign.^2^ In other words, the Continental Airlines welcoming formula is a common Hebrew phrase, both in Jesus’ times and in our own, and the hieratic connotations that we attribute to the ecclesiastical chant are by no means inherent in the words themselves, but have become identified with them over time in a particular type of religiously authorized use.

It follows that a correct pragmatic interpretation of such formulae should take into account both the circumstances of use and the way this use has developed. When the Jerusalem Jews, on the Sunday before that fateful *Pesach*, chanted ‘Blessed he who comes in the name of the Lord’, they were using a simple form of welcoming expression, one that is in use even in the Hebrew language today. And (to consider another Biblical instance of the same phenomenon), when Jesus talks about himself as the ‘Son of Man’, this is no exalted reference to some higher qualities he might possess in virtue of his being human, but simply a way of referring to himself by way of a modest ‘third person’ (in the same way as the Czechs may refer to themselves by the substantive *člověk*, as in *člověk nikdy neví* ‘one never knows’. In much the same way, the Germans utilize their ‘universal pronoun’ *man* as in *Man weiß nie*, similar to the Norwegian use of *en*, as in *en vet aldri*; other languages (such as Italian or Spanish) use an impersonal third person reflexive verb: *non si sa mai*, *nunca se sabe* ‘You never know’.

## Historical truth and linguistic facts III: grammaticalization

### Whatever happened to Latin?

The last example I quoted above demonstrates yet another tendency that is of interest to pragmatics, seen in an historical perspective: that of grammaticalization (or grammaticization, as it is also called). The famous French comparative linguist Meillet (1866–1936) has described this phenomenon as follows:

“Des mots, d’abord autonomes, se réduisent par l’usage à n’être plus que des éléments grammaticaux: en latin, *habeo* avait toute sa valeur dans *habeo aliquid factum*; mais, par l’effet de la répétition, *j’ai* de fr. *j’ai fait* a perdu progressivement toute autonomie; trois termes autrefois indépendants (*ego*, *habeo* et *factum*) qui ont abouti à fr. *j’ai fait* ne constituent plus aujourd’hui qu’une forme grammaticale équivalente à lat. *feci* et qui n’a pas plus de valeur expressive. **Les mots deviennent ainsi des éléments grammaticaux**,…”

‘Originally autonomous words, in the course of their usage, are becoming just grammatical elements: in Latin, *habeo* had all of its value in *habeo aliquid factum*; but, by dint of repetition, *j’ai* in the French *j’ai fait* has gradually lost all autonomy; the three erstwhile independent terms (*ego*, *habeo* et *factum*) that have resulted in French *j’ai fait*, today no longer constitute anything but a grammatical form that is equivalent to the Latin *feci;* it has no longer any expressive valor. **In this way, words become grammatical elements**,…’ (*Introduction,* p. 20; italics original, boldface mine)

It is well known (as Meillet underscores several times in the work quoted here) that languages are very different with regard to morphological complexity. Even within the same family, we see immense variations over time and space, from morphologically complicated languages like Sanskrit, where a single verb may have more than 350 distinct forms, to English, where the role of morphology in syntax has been reduced to an almost ‘Chinese-like’ level.

Similarly, there is a great variation among different languages as to the number and specifications of certain word classes; for instance, while verbs and nouns are properly distinguished in a language like Latin, the distinction is more blurred in English, such that verbs can become nouns and (more frequently) nouns may become verbs at a moment’s notice, so to speak (cf. ‘to OD on something’, from the substantive ‘overdose’, abbreviated ‘OD’).

The next section will deal with a particular morphological phenomenon, that of case and its descriptive vagaries in Indo-European.

### The case of case

The most common cases of grammaticalization occur in connection with the rise and fall of case systems. As we know, some languages excel in cases (some Caucasian languages, such as Lak or Tabassaran, sport up to fifty of them; Finnish has at least 14, maybe even 16, depending on how one counts them). Other languages, especially the older ones in the Indo-European family, have more modest, although still impressive inventories: Sanskrit with its eight, most Slavic language with their seven, Latin with its six, and Greek with its five cases are among the best known instances. But even a modern language such as German still can boast of having four cases, something which seems like a quaint anomaly in this neck of the linguistic woods, where all its close relatives and neighbors have shed the casual garb a long time ago (there are remnants of cases in both English, Dutch, and the Scandinavian languages, but these represent isolated or ‘petrified’ forms, the so called ‘genitive’ -*s* being among the former, the ‘dative’ ending -*e*, as in the Danish *i levende live* ‘in living [actual] life’, among the latter.

Above, I put the names of those ‘cases’ in scare quotes, meant to alert my readers to the fact that there was something uncommon in the way I was using the terms ‘genitive’ and ‘dative’). The next section explains why.

### Case as a category: a structuralist *katzenjammer*

During my linguistic apprenticeship in Copenhagen under the direction of the celebrated Louis Hjelmslev (1899–1965), one of the most zealously defended tenets of the extreme structuralist brand of linguistics known as ‘glossematics’ was that one never should indulge in ‘squinting grammar’. This term, whose origin goes back to another famous Danish linguist, Otto Jespersen, was used to methodologically disqualify those linguists who looked at one language when describing another. Typically, this was the case in what used to be called ‘school grammar’, where the Latin terms for the various cases (nominative, accusative, and so on) were uncritically transferred to languages that either had no such morphological formations (or at most, very few). This was how the English, Scandinavian, or German substantive forms ending in -*s*, came to be called ‘genitives’, whereas pronominal forms such as English *me*, Danish *mig*, Norwegian *meg*, German *mir* and *mich* could be described as either datives or accusatives, inasmuch as they corresponded to the Latin and Greek forms with the same function: *mihi* or *me*, *(e)moi* or *(e)me*.

Now what was wrong, according to the structuralists, with naming a case that functioned as a dative, a ‘dative’, and one that functioned as an accusative, an ‘accusative’? After all, as they say in Texas, “if it looks like a snake, moves like a snake, and strikes like a snake, then it probably *is* a snake”. However, the structuralists didn’t see it that way.

According to the axiom adopted by glossematics as well as by other schools of structuralist linguistics, there should be a strict correspondence between content and expression. Thus, a linguistic unit should be marked morphologically as a case in order to qualify for case status. Conversely, if a linguistic unit shows ‘case-like’ behavior (e.g. in its morphology or syntax) and can be assigned a ‘case-like’ content, then it has to be included among the number of cases in the language. If this two-way correspondence of content and expression was not in place, then we could not bestow the respective structural label on the linguistic unit in question.

### Inventorying case

The strict structuralist position results in some strange case inventories (for languages that do have cases, in the sense defined above) and some uncomfortable alternatives for those who want to obey the structuralists’ strictures in describing a language. As to the former, consider the inventory for Latin that we, Hjelmslev’s students in the fifties, arrived at during the seminars on ‘case’ [mostly based on Hjelmslev’s account of the phenomenon in his two volumes from the mid-thirties, *La catégorie des cas I & II*; Hjelmslev (1934, 1936)]. For Latin, the rules prescribed the inclusion of such rare forms as *statim* ‘immediately’ (from *status’* ‘stood’ (participle), *separatim* ‘apart from’ (from *separatus* ‘separated’), or *passim* ‘everywhere’ (from *passus* ‘step’). Here, as everywhere, a minor problem was how to give such isolated occurrences a proper ‘casual’ name (e.g. an ‘expansive’?); much worse was our agony over how to distinguish forms like *passim, statim* from bona fide adverbs of time and place (that, incidentally, also at one time might have been cases, cf. *cito* ‘quickly’, presumably the ablative of *citus*—but then what about *porro, retro, frustra* and all the other adverbs ending in long -*a* or-*o*?)

However, such objections were never taken seriously by the Master, and I even got a maybe deserved chiding in front of the class for my obstinacy and stubbornness, when I suggested that there was something wrong with a reasoning that would have us consider regular adverbial forms like Latin *unde*’ from where?’, *inde* ‘from there, then’, *deinde* ‘from there, since then’, and so on, as obscure ‘cases’, related somehow to the deictic or determinative pronoun *is* ‘the one there, the one just mentioned’. Or to take an example from Finnish: should *ohitse* ‘in passing’ be called an adverb (from *ohi* ‘past’), inflected for case? But adverbs are normally not inflected, so…^3^ On the other hand, Finnish *(i)tse* is itself a (semi-)productive suffix: when added to a substantive, it produces an adverbial expression indicating the way along which something is happening; well-known examples are *lentoteitse* ‘by way of air’ (from *lento* ‘flight’ and *tie* ‘road’; the equivalent of ‘By Air Mail’ on envelopes), *puhelimitse ‘*by phone’ (from *puhelin* ‘phone’), etc. But how to determine whether this a nominal case formation or an adverb?^4^

### Of snakes and cases

To return to the matter of grammaticalization: when a linguistic form changes its role, or adapts itself to a previous, now defunct or dying, function by incorporating new material, should we then not allow the old appellation to remain intact? It strikes like a snake, so it is a snake; it functions as a dative, so it is a dative, no? In this vein, one could talk about a dative function in a language that no longer has (or never had) a dative, a bit like the roles that Fillmore developed in his early version of case grammar, later more fully developed in all kinds of functional grammatical systems. A construction such as the Hindi N (or Pron) + *ka* (as in *ap ka gulam hai* ‘I am your servant’, (*ap*, respectful second person address form + ‘genitive’ particle −*ka*) would then be just as much of a genitive as the English N + *‘s* or the corresponding Scandinavian ‘genitives’—not to speak of the German so-called ‘*zweiter Fall*’ (lit. ‘second case’)—in reality a pseudonym for the old term ‘genitive’).

But the protean character of linguistic entities shows itself not only in this type of strict grammaticalization (‘the case of case’); linguistic units change form, function, or both at the same time in many other ways as well. The next section will deal with some functional changes.

## Changing functions, changing forms: the definite article

For a pragmaticist, the strict morphological requirements of a case inventory in the structuralist tradition have very little to do with the way a language functions. Similarly, pragmatics prefers to define linguistic categories and the changes that they incur over time, using functional, not morphological criteria. Let me show you some examples of a category which is famous for its protean character: the article (definite or indefinite).

### The Indo-European definite article

The word class ‘article’ is absent in a number of Indo-European languages such as Latin, Lithuanian, and the majority of the Slavic languages. The same is true for a number of unrelated languages such as Japanese, Chinese, or Greenlandic Inuit; by contrast, it flourishes in Indo-European languages such as Greek, German, the Romance descendants of Latin, English, and so on. What is interesting is that this particular word class does not seem to be on the way to extinction or weakening; on the contrary, we see it emerge as a new development in various geographically and linguistically very distinct locations, as I will show (below, in three parts).

Starting out with an overview of the situation in a card-carrying ‘articulate’ language such as Classical Greek, I will examine the emergence of article-like categories of determiners in such unrelated languages as Czech and Finnish (with a little excursus into Chinese).

### Part one: the classics

Right away I hear somebody protesting: But aren’t Czech, Finnish, and Chinese supposed to have no articles at all? And as for Greek itself, aren’t those so-called articles ‘really’ some kind of determinative, or even demonstrative pronouns?

With regard to Ancient Greek, the objection is valid: the article *ho* of the classical period is, historically, the same as the determinative/demonstrative *hos*; in the Homeric epics, the two are not clearly marked off against each other. Whenever needed, the demonstrative does duty (even if rarely, by our standards) as a definite article, or even as a relative pronoun.

For example, we find *Tá en Pyloi* ‘the things that happened in Pylos’ as the title of book 3 of the Odyssey; this *tà* is not really different (except functionally) from the *tà* used as the neuter plural article, as in *tá t’álla* (‘in other respects’, Zeus speaking to Hermes in Od. 5 :29, or from the same *tà*, used as a relative pronoun, as in *heímata tá hoi póre dîa Kalypsó* (ibid. 320, 371, etc.) ‘the garments that the lovely Calypso had provided him [Ulysses] with’.

Compare now that the Latin language, geographically and culturally, and even (albeit more remotely) linguistically close to Greek, kept its independence, article-wise, for all of its millennial life during which it managed to do without those pesky holdouts. Only much later, after the Classical and Vulgar Latin periods, we see the emergence of something like an article; the Medieval philosophers apparently felt it was necessary from time to time to indicate definiteness when speculating about the notions of ‘to be’ or ‘being’, letting the Latin expressions *esse*, *ens*, often be preceded by the borrowed Greek definite article in its singular neuter form: *to ens, to esse*. And of course, in the classical language’s various successors, in the transition from Vulgar Latin to the modern neo-Latin dialects, a parallel development has created articles in all of them, albeit in different guises and constructions (French *le, la*, Spanish and Italian *lo, la*, with Portuguese coming full circle (almost) to the Classical Greek forms: *o, a.* (As to Romanian, it obeys the typological laws of its linguistic area, the *Sprachbund*, by putting the definite article at the end, cf. masc. -*ul* in *om*-*ul* ‘the man’).

### Part two: Slavic, especially Czech

As to the Slavic languages, with the exception of Bulgarian (which follows the areal imperative of its *Sprachbund* by letting the definite article follow the substantive, as do Romanian and Albanian), the rule is that there are no definite or indefinite articles. Speakers of languages such as Russian, Polish, Czech, Slovak and so on experience infinite hardships when they wish to express themselves in a Western language such as English, which sometimes does, sometimes does not, require the presence of (mostly a definite) article.

Even so, there have been tendencies towards introducing an article-like preposed morpheme in Czech as early as the sixteenth century, and the tendency continues today. In the nineteenth century Czech writer Jan Neruda’s immortal sketches from the Prague quarter called *Malá Strana*, in German *Kleinseite*, we meet two gentlemen, about whom the author says:

“Přede všemi však zůstanou mně nezapomenutelni mužové dva, ti se vryli v srdce srdce mého. Pan Ryšánek a pan Schlegl…. Ti dva se nemohli ani cítit*”.*

‘Above all, nevertheless, unforgettable remain for me two men, they [*ti*] have engraved themselves in my heart of heart[s]. Mr. Rysánek and Mr. Schlegl. The [*ti*] two could simply not stand each other”. (*Povídky malostranské*, Praha, Mladé Letá, 1960, p. 90; my translation)

Here, we see how, similar to what is the case in Homeric Greek, the demonstrative pronoun *ten* (sing. masc.; plur. masc. *ti)* is used, on the one hand where we would expect a relative pronoun (my translation ‘they’ is somewhat clumsy: using the relative pronoun ‘who’ would give a much better result), and on the other as a kind of preposed definite article (‘the two’).^5^

To take a contemporary occurrence of the same phenomenon, it has been pointed out that the Czech demonstrative *ten*, especially in combination with the superlative, is not only a possible, but indeed a necessary completion of the construction, as seen in the following two minimal pairs, due to the UCLA linguist Kresin (2001, 2005):

*To je to nejdůležitější*/*To je nejdůležitější

(‘That’s the most important [thing]’)

*To je ta nejdůležitější věc*/*To je nejdůležitější věc* (‘That’s the most important thing.’) (Kresin 2005: 1)

Susan Kresin, to whom I am indebted for the above examples, concludes her observations as follows:

“This [first] minimal pair indicates that in the syntactic construct *to je X,* in which X is a superlative adjective, *ten* functions as an obligatory device of nominalization. In other words, the use of *ten* is *grammatically determined* by the mere existence of this syntactic construct—an indication of its full grammaticalization in this particular context.” (2005: 1) my emphasis)

Compare also the following extract from a contemporary Czech novelist (as quoted in Kresin, ibid.):

*… má máma nekdy trojcila, jako by na celym svete chodila do rachoty akorát ona sama a ostatní se flákali a lezeli drzkou na slunícku. Jo, zacala byt taková, ze mela tu nejtezsí drínu, tu nejvetsí otrocinu za nejmín prachu.*

‘My mother sometimes carried on about how only she in the whole world had to go to work, and everyone else just bummed around and lay in the sun. She started to act as if she had the heaviest load, the greatest slave-work for the least amount of money.’ (Václav Dusek, *Tuláci*)

To this, I would like to add an observation from my own experience as a part-time speaker of Czech. When my wife and I lived in Prague in the sixties, my first job in the morning would be to go across the street from the dormitory where we lived, and get the indispensable *housky* for our breakfast. The manager of the small convenience store where I used to shop for these things, a Mr. Karel Vlk, would invariably greet me with the words

*A co si preje ten soudruh?*

literally translated as:

‘And what does the comrade wish for?’

This was of course in the days of the ‘third’ (some would call it the ‘fourth’) republic; today, Mr. Vlk’s successor would probably not address me as ‘comrade’, but as ‘gentleman’, *pán*, but that’s beside the point—the point being that he used the demonstrative pronoun *ten*, grammaticalized to the status and function of an article. A correct English translation would be: ‘What does the gentleman want?’; in Danish (or Norwegian), one would say, in the same fashion, “Hva(d) ønsker herren?”

On another occasion, the woman cashier in the Charles University *menza*, or cafeteria, having suffered through my complaints about the lousy food served there, replied with a characteristic Pragueian shrug (pointing her thumb in the direction of the military academy next door, which got their meals from the same outlet):

“*Ti generalové to zerou!”*

that is, literally, ‘The generals fucking eat it!’ (with a deprecating verb, Czech *zrát*, that lacks a strict English counterpart (cf. German *fressen*, Danish *æde*); again, the demonstrative *ti* is used as an article here).

Often, the emerging article used in this fashion still connotes something of the original certain distance from the speaker, as we saw in the previous example; compare also the angry remark made to my wife when she tried to sneak into a long line waiting for the always elusive *brambory* (potatoes):

“*Hele hele ta paní, fronta je tady!”*

‘Wait a minute the lady, the line begins here!’

### Part three: Finnish

In Finnish, the tendency to ‘de-functionalize’ the demonstrative follows roughly the same path as we have seen for Czech. The negative connotations attached to the demonstrative (or better, determinative) *se* reflect a general aversion to the use of pronouns when referring to persons of distinction, reserving them for reference to equals or lower-placed individuals. A similar tendency is found in other language communities; in my Dutch-speaking family, for example, it was absolutely forbidden to refer to Dad as ‘he’, and the Swedish restrictions on the corresponding form (specially when used as a form of address) are well-known. The earlier, German-inspired Danish military manuals contained explicit sanctions against privates addressing their superiors inappropriately in this way; conversely, the Copenhagen police force used to be able to clamp a person in jail for addressing an officer with the 3d person singular masculine *han*.

Early in my Finnish life, I was instructed by my friends never to ask to speak to, or identify, a person on the telephone using the determinative pronoun *se* (literally ‘this’ in the sense of ‘the one closest in the context, the one just mentioned’, etc.) Thus, one should not literally translate an English ‘identifying’ question such as ‘Is that Professor Itkonen?’ by saying *Onko se professori Itkonen?*, but just drop the offending pronoun and ask *Onko professori Itkonen?*, thereby also conforming with the grammarians’ preference for pronoun-dropping with verbs.

Here, the *vox populi* shows a tendency to the contrary: pronouns are routinely used with finite verb forms, even though not ‘strictly’ needed; this is by now pretty pervasive trend, at least in the spoken language. But even in older times, the use of *se* normally would impart a rather impolite flavor, as e.g. in *se vanha piiantaimi* ‘that old dried-out biddy’ (literally, ‘an ‘(old) maid cactus’; the expression is found in the classical popular comedy *Nummisuutarit* (‘The village cobblers’) by the nineteenth-century author Alexis Kivi.

Otherwise, the fate of Finnish *se* has taken it in a somewhat different direction from the corresponding Czech pronoun. Actually, *se* is now used across the board in the spoken language as a replacement for the ‘real’ pronoun *hän* ‘he/she’ (Finnish does not have grammatical gender, such that the pronoun *hän* is ‘born’ politically correct, corresponding to the English written form ‘s/he’). Compare the following stretch of informal dinner table conversation between parents and their son. The question is about a cousin who has been traveling, and whether he will remember to bring back some present for the 11-year old boy.

FATHER: lupasiko se tuoda sulle tuliaisia?

Did he promise to bring you some presents?

CHILD: 0 [=! nods].

FATHER: millaisia?

What kind?

CHILD: se sanoi että se keksii sitten.

He said that he will think of something later.

FATHER: keksii jotakin.

He will think of something.

(Adapted from Tryggvason 2006)

In this excerpt, we see how the pronoun that agrees with the verb in lines 5 and 8 is not the expected personal pronoun *hän*, but the determinative *se* (corresponding to Latin *is*), whereas in line 9, we meet the normal, default case of PRO-drop, when the father repeats the child’s assurance *se keksii*, but without any pronoun: *keksii jotakin*.

What are we supposed to make of this? Assuming that the pronoun *se* has its true determinative value, one could surmise that the first mention of the traveling cousin had something of the same negative tinge to it that was identified above (the father thinking of the cousin as a person not really interested in buying the boy a gift). However, the fluent use of *se* in the child’s reply and the father’s confirmation of this seem to indicate that the expressions with and without *se*, while equivalent in referential value, also are very close semantically and connotation-wise. The pronoun *se* has simply lost its independent denotational character in the colloquial use attested here; it simply functions as a substitute for the personal pronoun (in fact, using *hän* here would sound a bit pompous and over-correct, especially in the child’s reply, whereas the father, too, seems quite happy either using *se*, or go PRO-dropping and use nothing at all.

As to *se*, it is reduced from a ‘full’ pronoun to a substitute for a pronoun (which already itself had been reduced to semi-autonomous status; as Meillet remarks:

“… ces petits mots [*je, tu, elle,…*] n’existent plus d’une façon indépendante et ne se rencontrent qu’avec le verbe;…” (‘these small words do not exist any longer in an independent way and are not found except in connection with the verb”; Meillet 1937: 440).

In this way, *se* fully qualifies for ‘grammaticalized’ status according to Meillet’s definition of the process by which words, along with their autonomy, “lose their proper meaning and become grammatical tools” (ibid.).^6^

### A Chinese footnote

I am not familiar with Chinese, so the following remarks may serve as hints, rather than as observations or postulates. In contemporary Mandarin, as the Taiwanese linguist Shuanfan Huang has convincingly shown, the demonstrative pronoun has started to function as a way-stage towards the development of a definite article (which Chinese and European grammarians traditionally have asserted does not exist in Chinese). The context for this development, according to Huang, is “interactional referring”, which triggers an evolution of the distal demonstratives to full-blown definite articles, in fact a functional change that results in a true grammaticalization. As Huang remarks,

“it is those interactional meanings that form the foundation of the grammaticized uses of the developing definite article in spoken Chinese. In particular,… the definite determiner is found to regularly emerge in interactional contexts where the speaker has reason to believe the identity of a referent to be community shared knowledge s/he can exploit” (Huang 1999: 79)

What this means for us, referring back to the Czech and Finnish examples cited above, is one, that these developments so far only have taken place in the spoken language; the written standards as defended by the grammarians and the ‘keepers of the word’ still adhere to the traditional view that the languages mentioned do not possess articles (or other definite determiners) in regular, default usage. The interactional aspect that Huang cites for modern Mandarin is especially interesting because it gives us another motivation to consider the developments outlined here as truly *pragmatic* phenomena, developed in a context that is typically characterized by the interaction between users of language, rather than in discussions between linguists and grammarians.

## Conclusion

I started out by referring to the ‘life of words’ as a pragmatic matter, one that comprises, but not exclusively, a historical development in the sense of a ‘history of words’, or even of sounds. Here, it behooves us to reflect on the very term ‘comparative grammar’, as it has been traditionally understood as the study of the sound correspondences between various languages, in the perspective of drawing up family resemblances and genealogical ‘trees’.

The study of grammar, taken in its widest sense as the way to use language correctly and effectively, unites the grammarian and the pragmaticist in one common goal. This goal has not been pursued too assiduously, neither in the historical nor in the structural tradition, even though lately, typological studies have begun to fill the gap that had been left open by both comparativists and strict structuralists.

As to the historical dimension, in particular the motivation behind language changes such as semantic bleaching and grammaticalization, the subject was broached by Meillet in many of his works, but not pursued further by him. Still, questions of language development as an historical process cannot be separated from the ontological development of language in the individual human, an insight that Meillet already possessed over a hundred years ago, but to which others, including myself, have come only relatively late in life.

In a pragmatic perspective, studies of grammar, and (especially) of the users of grammar, can profit from asking questions such as why a particular language has developed the way it did, or how a particular development in a language has taken place. But I can also see why such questions were deemed anathema by most historical linguists, and how they were perverted by the structuralists to fit in with their preconceived notions of how language works, with labeling taking the place of understanding.

I recall the shock and awe I experienced sixty years ago, upon reading, in Meillet’s *Introduction*, how “every time a child begins to speak, he introduces some innovations” (1937: 19; my translation). At the time, I objected not only to the issue being raised, but to its being raised in this context, at that by a *savant* like Antoine Meillet, who was famous for his work in ‘serious’, that is, comparative linguistics. For me, as a burgeoning structuralist, children and their imagined innovations simply had no place in that universe of studies. But Meillet, on the same page 19 of his *Introduction*, unabashedly expands his views and clearly states that: “such innovations are the beginnings of language development and change”, and goes on to say:

“[l’enfant] ne reçoit pas des autres des procédés d’articulation:… il ne reçoit pas des paradigmes grammaticaux: il recrée chaque forme sur le modèle de celles qu’on emploie autour de lui,…”

‘[The child] does not inherit articulatory procedures or grammatical paradigms; he re-creates every form according to the model of the forms that people around him use,…’ (Meillet 1937: 19)

I can still trace the contours of the word ‘nonsense!’ that I wrote in the margin of my copy of Meillet’s book!

If Meillet were around in our times, he would definitely reject any theory built upon ‘innate categories’ or systems, be they phonetic or grammatical. One could not imagine a more consistent rejection of all forms of hypothetical constructs à la the Chomskyan ‘LAD’ than the one given by Meillet in the quotation above. For Meillet, as for Virgil, words develop, grow, and in fact live, *for a reason*; that is, they ‘are about things’ (*sunt verba rerum*). At the end of the day, words (and in general, languages) are anchored in their use by the users, not in hypothetical constructs by linguists. By writing this article, I hope to have made a modest contribution to a pragmatically-inspired understanding of this ‘life of words’, and of how our words have come to be, and are, about the things we associate with them.

## Footnotes

1 In 1985, a number of US diplomats were kept under siege in the Tehran embassy for many months and were only liberated after humiliating negotiations.

2 The Hebrew version of Matthew 21:9 has *Barukh ha-ba’* ‘Blessed he who comes’, a formula identical (except for the singular form) to the one used in the Continental Airlines and the Tel Qatifa greetings. Incidentally, the picture with the sign was brought by the Danish free tabloid *Urban* on March 3, 2006, in a totally different and inappropriate context.

3 And neither are particles, according to the strict historical-grammatical view. Gustav Meyer takes Homer to task for “erroneously” inflecting these suffixes: “er [Homer] flectiert irrtümlicherwise auch die Partikel” (*Griechische Grammatik*, Leipzig 1888, 2nd ed. p. 395). The instance Meyer criticizes here is Odyssey 2:47: *humîn toísdessi* ‘to y’all who are here’.

4 The number of cases in Finnish is subject to dispute, and depends on who makes up the inventory (cf. also above). Auli Hakulinen, in her article on Finnish in the Elsevier *Encyclopedia of Language and Linguistics*, 1st ed., Amsterdam and Oxford, 1994, speaks of 14, maybe 15 cases; Fred Karlsson, in the second edition of OUP’s *International Encyclopedia of Linguistics*, lists only 13.

5 The translation ‘these two’ would be possible as well, but strikes me as maybe a little too emphatic.

6 Meillet does not mention the term ‘grammaticalization’ here, but the tendency is clear. Compare also another quote from Meilllet, describing grammaticalization as “l’attribution du caractère grammatical à un mot jadis autonome” (‘the attribution of grammatical character to a formerly autonomous word’; Meillet 1958). The quote is found in Hopper and Traugott (2003: 19); see the review by Visconti (2006).
